# Bruton’s tyrosine kinase inhibition limits endotoxic shock by suppressing IL-6 production by marginal zone B cells in mice

**DOI:** 10.3389/fimmu.2024.1388947

**Published:** 2024-04-04

**Authors:** Kazuhiko Kawata, Shinya Hatano, Akemi Baba, Keisuke Imabayashi, Yoshihiro Baba

**Affiliations:** Division of Immunology and Genome Biology, Medical Institute of Bioregulation, Kyushu University, Fukuoka, Japan

**Keywords:** Bruton’s tyrosine kinase (Btk), acalabrutinib, marginal zone B cell (MZB), sepsis, IL-6, TLR4, NF-κB, humoral immunity

## Abstract

Sepsis is a systemic inflammatory response to a severe, life-threatening infection with organ dysfunction. Although there is no effective treatment for this fatal illness, a deeper understanding of the pathophysiological basis of sepsis and its underlying mechanisms could lead to the development of new treatment approaches. Here, we demonstrate that the selective Bruton’s tyrosine kinase (Btk) inhibitor acalabrutinib augments survival rates in a lipopolysaccharide (LPS)-induced septic model. Our *in vitro* and *in vivo* findings both indicate that acalabrutinib reduces IL-6 production specifically in marginal zone B (MZ B) cells rather than in macrophages. Furthermore, Btk-deficient MZ B cells exhibited suppressed LPS-induced IL-6 production *in vitro*. Nuclear factor-kappa B (NF-κB) signaling, which is the downstream signaling cascade of Toll-like receptor 4 (TLR4), was also severely attenuated in Btk-deficient MZ B cells. These findings suggest that Btk blockade may prevent sepsis by inhibiting IL-6 production in MZ B cells. In addition, although Btk inhibition may adversely affect B cell maturation and humoral immunity, antibody responses were not impaired when acalabrutinib was administered for a short period after immunization with T-cell-independent (TI) and T-cell-dependent (TD) antigens. In contrast, long-term administration of acalabrutinib slightly impaired humoral immunity. Therefore, these findings suggest that Btk inhibitors may be a potential option for alleviating endotoxic shock without compromising humoral immunity and emphasize the importance of maintaining a delicate balance between immunomodulation and inflammation suppression.

## 1 Introduction

Sepsis is a life-threatening systemic inflammatory response (SIRS) to infection, leading to possible organ failure and death (1). Sepsis is the primary cause of death in most intensive care units (ICUS), warranting early detection and appropriate treatment. A better understanding of the pathophysiological basis of sepsis and the mechanisms underlying the inflammatory response will contribute to the development of new therapeutic strategies for this extremely fatal disease. Lipopolysaccharides (LPS) or endotoxins are the major cell wall components of gram-negative bacteria that are pivotal for the development of septic shock (endotoxin shock) (2). LPS signaling through Toll-like receptor 4 (TLR4) causes the release of cytokines and other inflammatory mediators from host cells, particularly from monocytes and macrophages. A recent study has indicated that pro-inflammatory IL-6 produced by marginal zone (MZ) B cells contributes to endotoxic shock (3). Two primary pathways are activated in LPS-stimulated cells depending on the adaptors involved: an early myeloid differentiation factor 88 (MyD88) pathway and a delayed MyD88-independent pathway (4). The MyD88-dependent pathway induces the early activation of nuclear factor-κB (NF-κB), which leads to the production of pro-inflammatory cytokines, and the MyD88-independent pathway induces the activation of interferon (IFN) regulatory factor 3 (IRF3) and the late activation of NF-κB, which leads to the production of type I IFN and IFN-stimulated genes (4). Hence, modulation of the TLR4 pathway is a promising strategy for the treatment of sepsis.

Bruton’s tyrosine kinase (Btk) is a member of the Tec family of tyrosine kinases that plays a crucial role in regulating B cell development (5). Mutations in the Btk gene lead to X-linked agammaglobulinemia (XLA) in humans, which results in almost complete inhibition of B cell maturation (6, 7). Btk is also involved in murine X-linked immunodeficiency (XID), in which a mutation in the pleckstrin homology domain results in impaired B-cell differentiation, although not as severely as in XLA (6–8). Btk plays a major role in signaling through the B-cell receptor (BCR) (9). Upon BCR ligation, Btk activates phospholipase C (PLC)-γ2 to produce inositol 1,4,5-trisphosphate (IP3) and diacylglycerol (DAG). This results in calcium release/influx and activation of PKC, respectively, and the induction of several downstream signaling pathways, including the NF-κB pathway (10). While several studies have suggested that Btk contributes to innate immunity and BCR signaling, whether TLR signals activate Btk in B cells remains unknown. Btk is expressed in various myeloid cell types and can be triggered in certain TLR responses, including TLR2 or TLR4 in human monocytes and macrophages (11, 12) and TLR2, TLR4, TLR7, and TLR8 in murine and human dendritic cells (13, 14).

In macrophages, activated Btk interacts with MyD88 and TRIF to activate pathways that depend on these molecules (15). Btk-deficient mice show reduced recruitment of M1 macrophages following LPS injection and impaired production of pro-inflammatory cytokines following LPS stimulation (16). Bone marrow-derived macrophages (BMDMs) lacking Btk showed reduced IL-10 production when stimulated with multiple TLR ligands, whereas IL-6 was enhanced (17). Notably, Btk-deficient mice are sensitive to endotoxic shock because of impaired B1a cell-derived IgM antibodies (18, 19). When Btk-deficient mice were reconstituted with purified normal mouse IgM, endotoxin lethality was restored to levels observed in normal mice. Therefore, whether Btk plays a crucial role in endotoxic shock and whether it could be a potential therapeutic target remains to be determined.

Acalabrutinib is a remarkably efficacious small-molecule inhibitor of Btk and has been approved for treating various types of blood cancers. This second-generation Btk inhibitor is highly specific to Btk and has minimal effects on other kinases (20–24). Acalabrutinib, which is a first-in-class drug, irreversibly binds to cysteine 481 (Cys481) at the Btk-ATP-binding site, preventing the autophosphorylation of tyrosine 223 (Y223) and blocking downstream BCR signaling. This mechanism is similar to that of ibrutinib, which inhibits interleukin 2-inducible T-cell kinase (Itk) in addition to Btk, raising concerns about unwanted effects on T cells. In contrast, acalabrutinib is more specific to Btk. Additionally, acalabrutinib is more potent than ibrutinib with faster oral absorption and a shorter half-life, which improves the safety and efficacy over first-generation Btk inhibitors (25). Originally, Btk inhibitors were used for cancer treatment but have recently been used to effectively treat inflammatory diseases (26–28). Despite the promising results in preclinical and early clinical studies (26), these inhibitors may increase the risk of secondary infections and decrease humoral immunity.

In this study, we aimed to understand the effects of acalabrutinib on LPS-induced inflammation and its potential immunocompromising side effects. Our findings show that acalabrutinib suppresses LPS-induced endotoxic shock *in vivo*. Although acalabrutinib exerts little effect on follicular B cells and macrophages, it shows remarkable inhibition of NF-κB activation and IL-6 production in MZ B cells. Moreover, short-term treatment with acalabrutinib did not affect the number of mature B cells or antigen-specific antibody responses. These results provide valuable information for developing therapeutic strategies against sepsis and understanding of the potential role of Btk in the inflammatory response.

## 2 Materials and methods

### 2.1 Mice

C57BL/6J mice were purchased from CLEA Japan (Japan). Btk KO mice were generated by the CRISPR/Cas9 system (**Supplemental Methods**). Mice were given pathogen-free conditions, and all studies and procedures were approved by Kyushu University Animal Experiment Committee. All animal experiments were conducted in accordance with ARRIVE guidelines and the ethical guidelines of Kyushu University.

### 2.2 Flow cytometry analysis

For flow cytometry, single-cell suspensions prepared from BM, spleen, and PEC were blocked by using FcR-blocking reagent (TONBO) and stained with fluorochrome-conjugated antibodies (**Supplementary Table 2**) and streptavidin (BioLegend). Dead cells were stained with 1 μg/ml propidium iodide (Nacalai Tesuque). Stained cells were analyzed on a Cytoflex (Beckman Coulter).

### 2.3 Cell sorting and B cell isolation

Single-cell suspensions were isolated from spleen and PEC. MZ B cells, FO B cells and Mac were sorted using FACSMelody (BD Biosciences). Cells were blocked by FcR-blocking reagent (TONBO) and stained with the following fluorochrome-conjugated antibodies (**Supplementary Table 2**) and streptavidin (BioLegend). For B cell isolation, splenic B cells were purified by negative selection of CD43^+^ cells with anti-CD43 magnetic beads (Miltenyi Biotec). The enriched B cell population was >95% positive for CD19 staining.

### 2.4 Immunization and enzyme-linked immunosorbent assay (ELISA)

Mice were given i.p. with 100 μg of NP-CGG in alum or 50 μg NP-Ficoll. For secondary immunization, mice were given i.p. with 100 μg NP-CGG without adjuvant. Mice were treated with acalabrutinib diluted in drinking water (**Supplementary Methods**). Levels of NP-specific antibodies were analyzed on 96 well plates coated with NP (29)-BSA (Biosearch Technology) and detected with HRP-conjugated goat anti-mouse IgM, IgG1, IgG3 (Southern Biotech). The amount of IL-6 in the culture supernatant was measured by ELISA according to the manufacturer’s protocol (BioLegend).

### 2.5 Experimental LPS-induced endotoxic shock

Mice were given i.p. injection of PBS or 20 mg/kg LPS (O55:B5, Sigma-Aldrich) dissolved in PBS. Mice were given p.o. vehicle or 10 mg/kg acalabrutinib (**Supplementary Methods**) at 1 h and every 12 h up to 3 days after LPS injection, and the mortality of mice was monitored.

### 2.6 Generation of bone marrow-derived macrophages (BMDM)

The BM cells were extracted from C57BL/6J mice by flushing femurs and tibias, and 4 x 10^5^ BM cells were cultured for 7 days at 37°C CO_2_ incubator using BMDM culture medium (**Supplementary Methods**).

### 2.7 Cell stimulation

1 x 10^5^ cells of MZ B cells and FO B cells from spleen, splenic Mac, and PEC Mac were stimulated with 10 μg/ml LPS (O55:B5, Sigma-Aldrich) for 6 h or 24 h in 96 well plate at 37°C in the presence of vehicle or 1, 10 μM acalabrutinib or, 1 μM BAY 11-7082 (Selleck Chemicals). CD43^-^ B cells were labeled with 20 μM Cell Trace Violet (Invitrogen) for 5 min at R.T. The cells were stimulated with anti-IgM F(ab’)_2_ (Jackson), anti-CD40 (HM40-3, BD Biosciences), recombinant mouse IL-2 (R&D), IL-4 (R&D), IL-5 (R&D) for 72 h or 96 h in 48 well plates at 37°C in the presence of vehicle or 1, 10 μM acalabrutinib. Acalabrutinib was dissolved in DMSO and diluted with culture medium (**Supplementary Methods**).

1 x 10^5^ cells of BMDM were stimulated with 1 μg/ml LPS (O55:B5, Sigma-Aldrich) and 20 ng/ml recombinant mouse IFN-γ (BioLegend) for 6 h or 24 h in 96 well plates at 37°C in the presence of vehicle or 1, 10 μM acalabrutinib.

### 2.8 Quantitative RT‐PCR analysis

Total RNA was isolated and purified using the RNeasy micro kit (Qiagen) from FO B cells, MZ B cells, splenic Mac, PEC Mac, and BMDM. cDNA was generated using the ReverTraAce qPCR RT Master Mix (TOYOBO). Real-time PCR was performed on a LightCycler 96 (Roche) using Thunderbird SYBR qPCR mix (TOYOBO). The primer sets used in this study were listed in **Supplementary Table 3**. The data were normalized to an *β-actin* reference.

### 2.9 Immunoblotting

Immunoblotting sample preparation was described in **Supplemental Methods**. Samples were electrophoresed into SDS-polyacrylamide gel and transferred to 0.45 μm PVDF membranes (Merck Millipore) by electrophoresis. Membranes were blotted with primary antibody and HRP-conjugated second antibody (**Supplementary Table 4**). For protein visualization, Chemi-Lumi One (Nacalai Tesque) or ECL Prime Western Blotting Detection Reagents (Cytiba) were used as substrates and protein levels were detected using a FUSION SOLO S (VILBER).

### 2.10 Statistical analysis

GraphPad Prism 8 (GraphPad Software) was used for all statistics. Unpaired student’s *t* test and two-tailed Welch’s *t*-test were used when only two groups were compared, and one-way ANOVA/Tukey was used for multiple comparisons. p values < 0.05 were considered to represent significant differences.

## 3 Results

### 3.1 Acalabrutinib alleviates LPS-induced inflammation and endotoxic shock

We first determined the inhibitory effects of acalabrutinib on Btk and its downstream signaling pathways *in vitro* and *in vivo*. A previous report showed that acalabrutinib binds covalently to Cys481 in the ATP-binding pocket of Btk at 1 μM concentration (21). Our study revealed that acalabrutinib efficiently blocks Btk autophosphorylation at Y231 in naive murine splenic B cells treated with a lower concentration of 0.1 μM acalabrutinib (**Supplementary Figure 1A**). Acalabrutinib significantly inhibits the phosphorylation of its direct substrate PLC-γ2 without affecting the phosphorylation of its non-substrate Erk (**Supplementary Figure 1A**). Acalabrutinib also suppressed increased expression of B cell activation markers CD69, CD80, and CD86 on FO and MZ B cells upon B cell receptor (BCR) stimulation (**Supplementary Figure 1B**). Furthermore, we investigated the inhibitory effect of acalabrutinib on Btk *in vivo* when administered orally (PO) via gavage at a single dose (10 mg/kg body weight) for 3 h (**Supplementary Figure 1C**). B cells from mice treated with acalabrutinib showed impaired BCR-induced CD69, CD80, and CD86 surface expression (**Supplementary Figure 1D**) and Ca^2+^ mobilization on FO and MZ B cells (**Supplementary Figure 1E**). These results suggest that acalabrutinib efficiently inhibits Btk activity both *in vitro* and *in vivo*.

**Figure 1:**
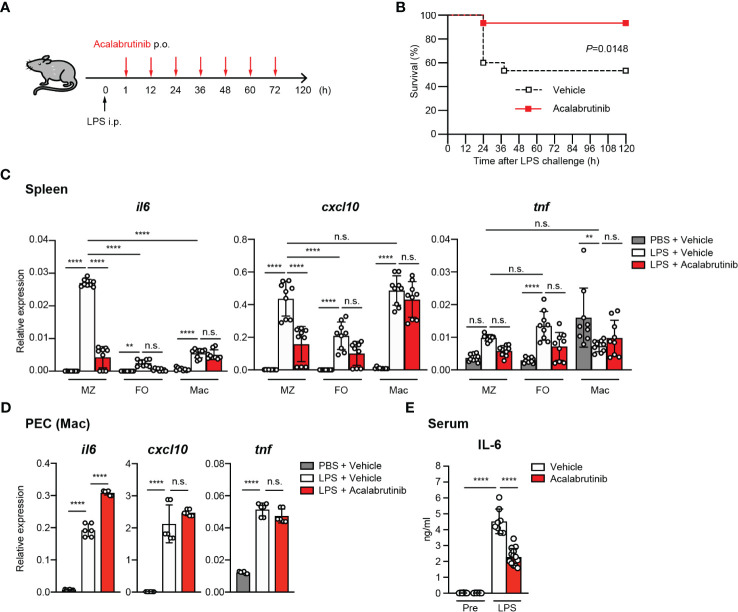
Acalabrutinib inhibited LPS-induced inflammation and endotoxic shock. **(A)** Experimental design for the LPS model. Mice were given intraperitoneal (i.p.) injection of PBS or 20 mg/kg LPS, then vehicle or 10 mg/kg acalabrutinib were treated by per os (p.o.) at 1 h and every 12 h up to 72 h after LPS injection. **(B)** Survival curves of mice treated with acalabrutinib and vehicle. Statistical analyses of survival curves were performed by the log-rank test. Statistically significant differences are shown (*P* = 0.0148). Data are presented from fifteen mice per group. Quantitative RT-PCR of mRNA encoding of *il6, cxcl10*, and *tnf* in marginal zone B cells (MZ), follicular B cells (FO), or macrophages (Mac) from spleen **(C)** or peritoneal cavity (PEC) **(D)** 6 h after injection of 20 mg/kg LPS. **(E)** ELISA assay of IL-6 in serum of mice with LPS injection after 24 h with vehicle or acalabrutinib. Data shown are pooled from three **(B, C, E)** or two **(D)** independent experiments. Significant differences are shown (n.s., not significant, **p < 0.01 and ****p < 0.0001; using one-way ANOVA/Tukey).

To investigate the effects of acalabrutinib on LPS-mediated endotoxic shock *in vivo*, we injected mice with a lethal dose of bacterial LPS (20 mg/kg body weight) before administering either vehicle or acalabrutinib (**Figure 1A**). We found that the acalabrutinib-treated mice were highly resistant to LPS-induced mortality (**Figure 1B**). To investigate how acalabrutinib controlled mortality, we examined its effect on the production of pro-inflammatory cytokines in MZ and FO B cells and macrophages isolated from LPS-injected mice (**Supplementary Figures 2A, B**). It should be noted that the number of splenic B cells and macrophages was not altered after acalabrutinib administration (**Supplementary Figures 2A, C**). We found that *il6* and *cxcl10* mRNA expression, which significantly increased in the MZ B cells in the spleen of LPS-injected mice, was pronouncedly suppressed by acalabrutinib treatment. In contrast, *tnf* expression was not inhibited by acalabrutinib. Although expression of *il6*, *cxcl10*, and *tnf* was enhanced in splenic FO B cells, acalabrutinib failed to suppress their expression (**Figure 1C**). Splenic macrophages also showed upregulated *il6* and *cxcl10* expression in LPS-injected mice. However, *il6* expression was lower compared to MZ B cells and acalabrutinib was ineffective in inhibiting *il6* and *cxcl10* expression (**Figure 1C**). In intraperitoneal macrophages, the administration of acalabrutinib failed to inhibit *il6*, *cxcl10*, and *tnf* expression (**Figure 1D**). Furthermore, acalabrutinib significantly reduced serum IL-6 levels in LPS-injected mice (**Figure 1E**). These findings suggest that acalabrutinib primarily suppresses endotoxic shock by limiting the expression of pro-inflammatory cytokine and chemokine in MZ B cells.

### 3.2 Acalabrutinib suppresses *in vitro* IL-6 produced by LPS-stimulated MZ B cells

**Figure 2:**
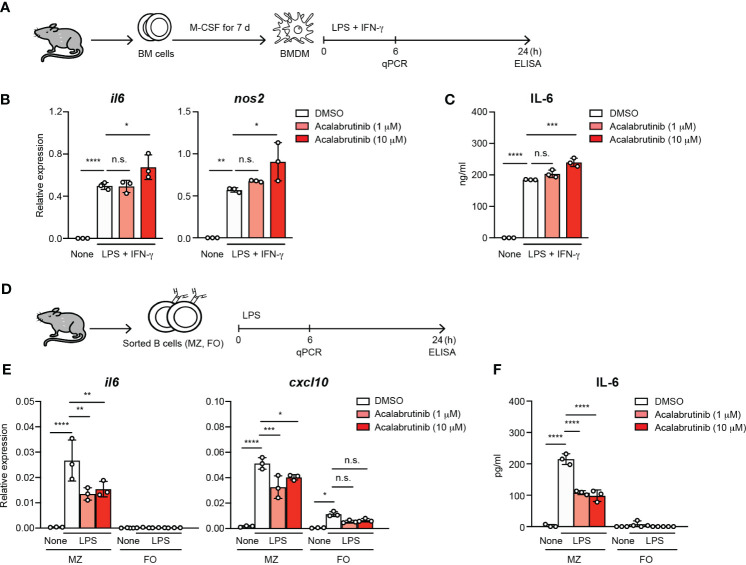
The treatment of acalabrutinib *in vitro* reduced the IL-6 secretion by MZ B cells. **(A)** Schematic of screen workflow. Bone marrow-derived macrophages (BMDM) was generated from bone marrow (BM) cells by culture with 10 ng/ml M-CSF for 7 days. The BMDM cultured with 1 or 10 μM acalabrutinib for 1 h, then were stimulated by 1 μg/ml LPS and 20 ng/ml IFN-γ including acalabrutinib for 6 or 24 h. **(B)** Quantitative RT-PCR of mRNA encoding of *il6* and *nos2* of BMDM 6 h after stimulation by LPS and IFN-γ with acalabrutinib. **(C)** ELISA of IL-6 secreted by BMDM after stimulation with LPS and IFN-γ with acalabrutinib for 24 h. **(D)** Schematic of screen workflow. Splenic MZ or FO B cells were incubated with 1 or 10 μM acalabrutinib for 1 h, then were stimulated with 10 μg/ml LPS in the presence of acalabrutinib for 6 or 24 h. **(E)** Quantitative RT-PCR of mRNA encoding of *il6* and *cxcl10* in MZ and FO B cells 6 h after LPS stimulation. **(F)** ELISA of IL-6 secreted by MZ and FO B cells after stimulation with LPS for 24 h. Data are presented as mean ± SD for three mice per group. Data are representative of three independent experiments. Significant differences are shown (n.s., not significant, *p < 0.05, **p < 0.01, ***p < 0.001, and ****p < 0.0001; using one-way ANOVA/Tukey).

To understand the direct effect of acalabrutinib on macrophages, we first investigated the responses of the bone-marrow-derived macrophages (BMDM) to LPS and IFN-γ, which are known as M1 polarization conditions, in the presence of acalabrutinib (**Figure 2A**). Acalabrutinib failed to inhibit the expression of *il6* and *nos2*, which indicates M1-macrophage-related phenotype, and the secretion of IL-6 (**Figures 2B, C**). Responses of MZ and FO B cells to LPS were examined *in vitro* in the presence or absence of acalabrutinib (**Figure 2D**). The LPS-stimulated upregulation of *il6* and *cxcl10* transcript levels in MZ B cells was substantially suppressed by acalabrutinib (**Figure 2E**). Secretion of IL-6 by MZ B cells was attenuated by acalabrutinib as assessed using ELISA (**Figure 2F**). These results suggest that acalabrutinib may target MZ B cells as cytokine-producing cells.

### 3.3 Btk-dependent LPS-activated NF-κB is required for IL-6 production by MZ B cells

**Figure 3:**
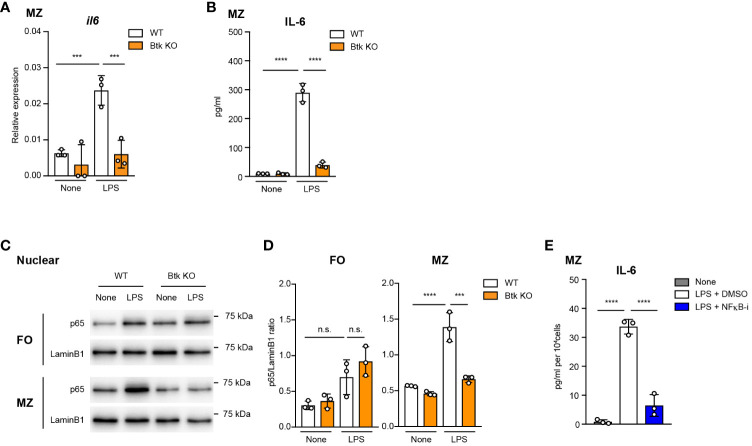
Btk-dependent NF-κB activation is required for IL-6 production from LPS-stimulated MZ B cells. **(A)** Quantitative RT-PCR of mRNA encoding of *il6* in MZ B cells from wild-type (WT) and Btk KO mice 6 h after 10 μg/ml LPS stimulation. **(B)** ELISA of IL-6 secreted by MZ B cells after stimulation with LPS for 24 h. **(C)** Immunoblot analysis of nuclear fraction lysates of sorted FO and MZ B cells stimulated with 10 μg/ml LPS for 60 min. **(D)** IL-6 in culture supernatant of MZ B cells was measured 24 h after 10 μg/ml LPS stimulation with or without 1 μM NFκB inhibitor (NFκB-i) by using ELISA assay. Data are presented as mean ± SD for three mice **(A-D)**. Data are representative of two **(A, B, D)** or three **(C)** independent experiments. Significant differences are shown (n.s., not significant, ***p < 0.001, and ****p < 0.0001; using one-way ANOVA/Tukey in **(A-D)**.

TLR4 stimulation through LPS in myeloid-lineage cells results in the activation of NF-κB, leading to the production of various pro-inflammatory cytokines such as IL-6 (29, 30). We investigated whether Btk regulates LPS-mediated IL-6 expression via NF-κB signaling in MZ B cells. As we showed in **Figures 2E, F**, acalabrutinib did not completely prevent the IL-6 production by MZ B cells, probably because it partially inhibits Btk. Therefore, to accurately assess Btk-dependent signaling, we generated Btk-knockout (Btk KO) mice (**Supplementary Figures 3A–C**). We confirmed that these mice have the same phenotype of B cell differentiation as previously reported (**Supplementary Figures 3D–F**, **Supplementary Table 1**). We found that mRNA expression and secretion of IL-6 was markedly reduced in Btk-deficient MZ B cells (75% reduction at the protein level) (**Figures 3A, B**), while acalabrutinib lowered it by only about half (**Figure 2F**). To evaluate the signaling pathway required for Btk activity, we isolated MZ B cells from Btk-deficient mice and examined LPS-stimulated NF-κB activation. Upon LPS stimulation, nuclear translocation of p65 was significantly observed in wild-type MZ B cells, but this was suppressed in Btk-deficient MZ B cells (**Figure 3C**). In FO B cells, however, nuclear translocation of p65 tended to be slightly increased but was neither statistically significant nor Btk-dependent (**Figure 3D**). Furthermore, the NF-κB inhibitor, BAY 11-7082, suppressed IL-6 production in MZ B cells (**Figure 3E**). These data suggest that the TLR4–Btk–NFκB signaling pathway is crucial for IL-6 production in MZ B cells.

### 3.4 Effect of acalabrutinib on B cell development and antibody immune responses

**Figure 4:**
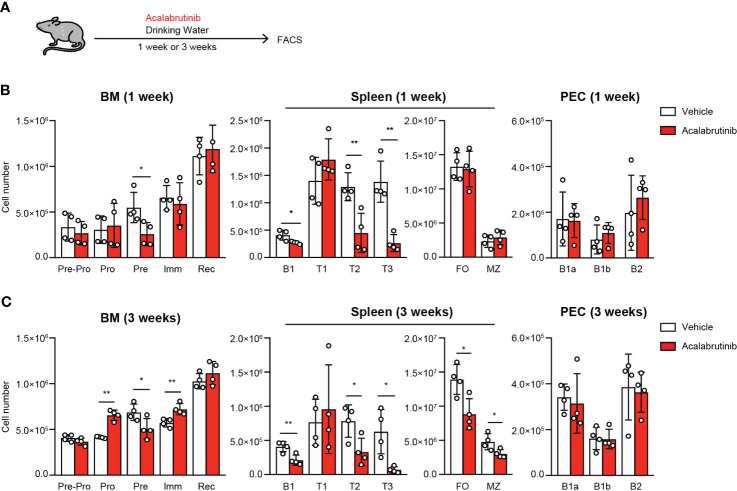
The effect of acalabrutinib on B cell development. **(A)** Schematic of screen workflow. Vehicle (2% HPBCD) or 0.15 mg/ml acalabrutinib were administrated by drinking water for 1 week **(B)** or 3 weeks **(C)**. **(B, C)** Flow cytometry of each B cell subset from BM, spleen, and PEC from mice treated with vehicle or acalabrutinib. B cell subsets were as follow: BM, Pre-Pro B cell (Pre-Pro; CD19^-^B220^+^), Pro B cell (Pro; CD19^+^B220^+^IgM^-^IgD^-^CD43^lo/+^CD25^lo/-^), Pre B cell (Pre; CD19^+^B220^+^IgM^-^IgD^-^CD43^lo/-^CD25^lo/+^), Immature B cell (Imm; CD19^+^B220^+^IgM^+^IgD^-^) and Recirculated B cell (Rec; CD19^+^B220^+^IgM^+^IgD^+^); spleen, FO B cell (FO; CD23^+^CD21^int^CD19^+^B220^+^AA4.1^-^), MZ B cell (MZ; CD23^lo^CD21^hi^CD19^+^B220^+^AA4.1^-^), Transitional 1 B cell (T1; IgM^hi^CD23^-^AA4.1^+^CD19^+^B220^+^), Transitional 2 B cell (T2; IgM^hi^CD23^+^AA4.1^+^CD19^+^B220^+^) and Transitional 3 B cell (T3; IgM^lo^CD23^+^AA4.1^+^CD19^+^B220^+^), B1 B cell (B1; CD19^+^B220^lo/-^); PEC, B1-a B cell (B1a; CD19^+^CD43^+^CD5^+^), B1-b B cell (B1b; CD19^+^CD43^+^CD5^-^) and B2 B cell (B2; CD19^+^CD43^-^CD5^-^). The number of cells was calculated on the basis of total cell count and flow cytometric analysis. Data are presented as mean ± SD for four mice per group **(B, C)**. Data shown are pooled from two independent experiments **(B, C)**. Significant differences are shown (*p < 0.05 and **p < 0.01 < 0.0001; using unpaired two-tailed Student’s *t*-test or two-tailed Welch’s *t*-test).

Acalabrutinib potentially increases the risk of impaired humoral immunity since Btk is essential for B cell development (5). To understand the potential adverse effects of acalabrutinib, we examined B cell development and antibody responses in mice that were administered acalabrutinib for an extended period of time through drinking water (**Figure 4A**). We observed a decrease in the bone marrow pre-B cells and spleen T2 cells after 1 week of treatment, but there was no change in the number of FO B cells and MZ B cells (**Figures 4B, C**; **Supplementary Figures 4A, B**). After 3 weeks of treatment, the Pro- to Pre-B cell ratio increased in the bone marrow, and the number of FO B and MZ B cells in the spleen decreased (**Figures 4B, C**; **Supplementary Figures 4D, E**). These results are similar to those observed in Btk-deficient mice with the only difference being that acalabrutinib did not affect B1 cells in the peritoneal cavity (PEC) (**Figures 4B, C**; **Supplementary Figures 4C, F**), which is a significant source of natural IgM antibodies (31).

**Figure 5:**
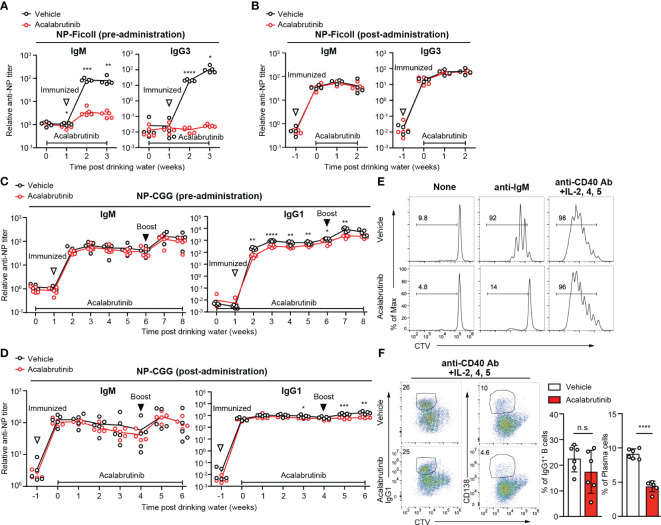
The effect of acalabrutinib on antibody responses. **(A, B)** NP-specific IgM and IgG3 antibody (Ab) in serum from mice immunized with NP-Ficoll were assessed by ELISA assay. Mice were treated with vehicle or 0.15 mg/kg acalabrutinib in drinking water 1 week before immunization **(A)** or 1 week after immunization **(B)**. **(C, D)** NP-specific IgM and IgG1 Ab in serum from mice immunized with NP-CGG in alum and boosted with NP-CGG 5 weeks after primary immunization were assessed by ELISA assay. Mice were treated with vehicle or acalabrutinib in drinking water 1 week before primary immunization **(C)** or 1 week after primary immunization **(D)**. **(E, F)** Proliferation and differentiation of B cells labeled with Cell Trace Violet (CTV) and stimulated as indicated with vehicle or 1 μM acalabrutinib. B cells were cultured for 3 days with no stimulation (None) and 10 μg/ml anti-IgM Ab, and for 4 days with anti-CD40 Ab, IL-2, IL-4, and IL-5. **(F)** Representative FACS plots and percentage of IgG1 class switch B cells and CD138^+^ plasma cells stimulated anti-CD40 Ab, IL-2, IL-4, and IL-5. The white-filled triangle indicates the immunization time, and the black-filled triangle indicates the time of booster immunization. Data are presented as mean ± SD for four to six mice **(A–F)**. Data shown are pooled from two independent experiments **(F)**. Significant differences are shown (n.s., not significant, *p < 0.05, **p < 0.01, ***p < 0.001, and ****p < 0.0001; using unpaired two-tailed Student’s *t*-test or two-tailed Welch’s *t*-test).

As it is believed that sepsis should be treated for a short period of time in the acute phase, it is assumed that acalabrutinib is administered for a short period of time. However, Btk inhibition is typically associated with the risk of immunodeficiency. To investigate the impact of acalabrutinib on antibody immune responses *in vivo*, mice were administrated vehicle or acalabrutinib via drinking water for a week before immunization with NP-Ficoll, which is a T cell-independent antigen (**Figure 5A**). We found that antigen-specific IgM and IgG3 antibody production decreased substantially following acalabrutinib treatment, although this reduction was not observed when acalabrutinib was administered 1 week after immunization (**Figure 5B**). The chronic extended use of acalabrutinib, particularly in individuals with chronic lymphocytic leukemia (CLL), may result in immunodeficiency, which is a risk factor for infections. To investigate the impact of long-term treatment of acalabrutinib, we next examined T cell-dependent (TD) antibody responses after immunization with NP-chicken-γ-globulin (CGG) and monitored the production of antigen-specific IgM and IgG1 antibodies (**Figure 5C**). We found that acalabrutinib did not affect IgM antibody responses regardless of whether acalabrutinib was administered 1 week before or after immunization (**Figures 5C, D**). However, mice treated with acalabrutinib 1 week before immunization showed a significant decrease in IgG1 antibody production (**Figure 5C**). In contrast, IgG1 primary responses were normal in mice treated with acalabrutinib 1 week after immunization, although secondary responses were slightly impaired (**Figure 5D**). Thus, long-term treatment with acalabrutinib may lead to a reduction in humoral immunity, but short-term treatment had no significant effect, suggesting that Btk inhibitors may alleviate endotoxic shock without compromising humoral immunity. It also implies that acalabrutinib treatment may not exacerbate the infection that caused the sepsis.

We next conducted *in vitro* B cell stimulation assays to study the direct effects of acalabrutinib on B cells. Acalabrutinib inhibited B cell proliferation induced by anti-IgM stimuli but not by anti-CD40 plus cytokines (**Figure 5E**). Class switch recombination was also not affected; however, plasma cell differentiation was partially inhibited by acalabrutinib following anti-CD40 antibody and cytokine stimulation (**Figure 5F**), suggesting that Btk may signal downstream of CD40 or cytokines for plasma cell development. Given that signals from BCR and CD40 in B cells are vital for antibody production, acalabrutinib may limit Btk activity and reduce antibody production.

## 4 Discussion

In this study, we investigated the immunological effects of therapeutic blockade of Btk with acalabrutinib. In an LPS injection model, acalabrutinib ameliorated clinical endotoxic shock and cellular inflammation. Importantly, IL-6 production by MZ B cells, which is critical for LPS-induced systemic inflammation, was suppressed by acalabrutinib treatment. By dissecting the clinical benefits, we found that short-term Btk inhibition prevented pro-inflammatory cytokines with little effect on B cell development or antibody responses. In contrast, prolonged treatment may carry the risk of lowering humoral immunity.

Btk inhibitors have been described as potential therapeutic agents for the treatment of sepsis (27, 32). For example, ibrutinib improved renal function in experimental polymicrobial sepsis caused by cecal ligation and puncture (CLP) (33). Recently, acalabrutinib was reported to reduce cardiac dysfunction caused by CLP (34). However, these studies either examined the effects of the inhibitors on macrophages or did not address the target cells of the inhibitors. In our study on LPS-induced systemic conditions, we demonstrated that acalabrutinib effectively ameliorated endotoxic shock and suppressed IL-6 production by MZ B cells. This is consistent with a previous study showing that IL-6 production by MZ B cells in LPS-injected mice causes endotoxic shock (35). Myeloid lineage cells, including macrophages and monocytes, are putative pro-inflammatory sources. A recent study indicated that acalabrutinib may be a valuable treatment for COVID-19 by inhibiting monocyte IL-6 production (36). While several reports have suggested that the inhibition of macrophage function by Btk inhibitors may improve disease pathogenesis (33), acalabrutinib exerted no discernable effect on IL-6 production by macrophages in this study, which is consistent with previous reports using Btk-deficient mice (17) or human samples (37). Additionally, Btk-independent ERK pathway plays a role in IL-6 expression macrophages (17). Our study, on the other hand, suggests that the NF-κB pathway regulates IL-6 expression in MZ B cells. Thus, it appears that MZ B cells and macrophages use distinct signaling pathways for IL-6 expression when exposed to LPS. Thus, these findings strongly suggest that acalabrutinib effectively treats septic shock by suppressing IL-6 production in MZ B cells. However, we cannot exclude the possibility that other factors expressed by macrophages or other myeloid cells are involved in endotoxic shock; for example, neutrophils and dendritic cells also express Btk, and its deficiency in these cells cause functional defects (13, 38). Hence, further studies are needed to determine the mechanism by which acalabrutinib inhibits endotoxic shock.

Btk is involved in TLR4 signaling in myeloid lineage cells; however, little is known about its role in B cells. Interestingly, this study shows that only MZ B cells, not FO B cells, activate NF-κB in response to LPS, and that this activation is dependent on Btk. The mechanism underlying the integration of Btk into the NF-κB signaling pathway in MZ B cells remains unknown. However, given that in myeloid cells, Btk interacts with four different proteins, TIR, MYD88, IRAK1, and TIRAP/MAL (11, 15, 39), downstream of TLR signaling, a similar molecular mechanism might be involved in MZ B cells. Further studies are required to elucidate the underlying mechanisms.

B1 cells, in contrast to B2 cells, protect against LPS- or CLP-induced septic shock through natural IgM antibodies or IL-10 production (18, 19, 40). Notably, Btk-deficient mice show fewer B1a cells and natural IgM in serum (31, 41) (**Supplementary Figure 3F**, **Supplementary Table 1**). Consequently, these mice are more sensitive to septic shock. In this study, we did not need to consider this effect on sepsis because mice treated with acalabrutinib had unaffected B1 cells. This finding is supported by another experiment regarding conditional Btk knock-out mice relying on tamoxifen-driven ERT2/Cre-mediated recombination (Btk cKO), in which the *Btk* gene was deleted after B cell populations were established (41). These mice showed normal B1a cells and natural IgM levels, in contrast to that observed in case of global Btk deficiency.

The study in Btk cKO mice revealed that Btk deletion in adult mice that had established B cell development did not affect B cell development in a steady state but rather affected B cell function (41). In the current study, acalabrutinib-treated mice exhibited lower IgM and IgG3 antibody levels during T-I response. This implies that mice taking acalabrutinib may have weaker antibody production against T-I antigens and a higher risk of related infections. T-I antibody response is critical for host defense against encapsulated bacteria with highly repetitive surface structures. In particular, *Streptococcus pneumoniae* (*S. pneumoniae*), which has a dominant polysaccharide capsule surface structure, plays a critical role in virulence principally by interfering with host opsonophagocytic clearance mechanisms. Treating patients with B-cell malignancies with other Btk inhibitors, such as ibrutinib, has been reported to cause severe adverse infectious events (42). Whether this effect occurs against bacterial infection, such as that of *S. pneumoniae*, remains unclear. Hence, caution should be taken when treating overwhelming inflammation with Btk inhibitors. In contrast, treatment with acalabrutinib after immunization did not affect the T-I immune response. This suggests that once immunity has been established, it is not affected by acalabrutinib treatment for a short period of time. This information can be helpful for determining to whom and when to administer the drug. In the case of TD response, which is the major immune response, we demonstrated that acalabrutinib administration reduced humoral immunity and memory responses only when administered over a prolonged period, and it did not completely inhibit humoral immune responses. Antigen-specific antibody responses are crucial for host defense against pathogens and for the prevention of infection by vaccines. Treatment with acalabrutinib immediately after infection is advantageous as a therapeutic agent because it suppresses sepsis and does not affect the immune response. However, another key finding of this study is that long-term drug administration, such as that required for patients with CLL, may lead to infection risks and vaccine inefficiencies. As an increasing number of Btk inhibitors are expected to be widely used to treat various diseases, including cancer, inflammation, cGVHD, and autoimmune diseases (25, 26, 33, 43), these data will provide helpful information for deciding therapeutic strategies.

## Data availability statement

The raw data supporting the conclusions of this article will be made available by the authors, without undue reservation.

## Ethics statement

The animal study was approved by Animal Experiment Committee of Kyushu University. The study was conducted in accordance with the local legislation and institutional requirements.

## Author contributions

KK: Writing – original draft, Writing – review & editing, Investigation. SH: Writing – original draft, Writing – review & editing, Investigation. AB: Writing – review & editing, Investigation. KI: Writing – review & editing. YB: Funding acquisition, Writing – original draft, Writing – review & editing, Conceptualization, Project administration, Supervision.
