# Supplementary material for: Bruton’s tyrosine kinase inhibition limits endotoxic shock by suppressing IL-6 production by marginal zone B cells in mice
Source: Front Immunol. 2024 Apr 4;15:1388947. doi: 10.3389/fimmu.2024.1388947 (PMC11024364; doi:10.3389/fimmu.2024.1388947)
Supplement: Supplementary file 1 [file DataSheet_1.pdf]

Supplementary Material

Supplemental Methods

Mouse

Btk KO mice were generated by the CRISPR/Cas9 system. Fertilized eggs were electroporated with 6 μg recombinant Cas9 Nuclease 3NLS (IDT#1074181), 0.5 μM crRNA (IDT; 5'-AGUUCAUUUUACACUACCACguuuuagagcuaugcu-3', 5'-CUCUGAUCCAAACACGGGAUguuuuagagcuaugcu-3') annealed with tracrRNA (IDT#1072532) in 1×Opti-MEM medium (Thermo Fisher) using NEPA 21 super electroporator (NEPA GENE) and 2 mm gap cuvette.

Culture medium

For BMDM culture: RPMI 1640 medium (Wako) supplemented with 10% (vol/vol) heat-inactivated FCS, 10 ng/ml recombinant murine M-CSF (Pepro Tech), 1 mM sodium pyruvate solution (Nacalai Tesuque), 100 U/ml Penicillin 100 μg/ml (Nacalai Tesque), and 10 mM HEPS buffer solution (Nacalai Tesque). For B cell culture：RPMI 1640 medium (Wako) supplemented with 10% (vol/vol) heat-inactivated FCS, 2 mM L-Glutamine (Nacalai Tesque), 1 mM sodium pyruvate solution (Nacalai Tesque), 100 U/ml Penicillin 100 μg/ml (Nacalai Tesque), and 10 mM HEPS buffer solution (Nacalai Tesque), 50 mM β-mercaptoethanol (Sigma Aldrich).

Drug treatment

For the treatment by per os (p.o.), acalabrutinib was dissolved with 0.5% Methylcellulose (Sigma-Aldrich) and 0.1% TWEEN 80 (Sigma-Aldrich) in water by using an ultrasonic bath sonicator (Branson). Mice were administrated 10 mg/kg acalabrutinib in one dose. For the treatment by drinking water, acalabrutinib was dissolved in 2% Hydroxypropyl Beta Cyclodextrin (HPBCD) and pass through 0.22 μm filter (Millipore). Mice were administrated 0.15 mg/ml acalabrutinib dissolved water via drinking water for 1 week or 3 weeks. For *in vitro* assay, acalabrutinib and BAY 11-7082 (Selleck Chemicals) was dissolved in DMSO (Wako). Cells were treated with 0.1, 1, or 10 μM acalabrutinib or 1 μM BAY 11-7082.

Genomic PCR analysis

Genomic DNA isolated from tail biopsies was used for PCR analysis. For *Btk* deletion, the following primer pair was used: sense primer 5'-ACTTGGGATGTAGCCTTCCTGC-3' and antisense primer 5'- GGCTTCATATAACTGGGTCCTCATACTG-3' (*Btk* deletion); sense primer 5'-

GTAATTTCCCTGTATACCTGATCTTCCTCTG-3' and antisense primer 5'-GGCTTCATATAACTGGGTCCTCATACTG-3' (wild-type).

Ca^{2+} measurement

For cytosolic Ca^{2+} concentration measurement, splenocytes were loaded with 0.5 μM Cal-520 (AAT Bioquest) and Pluronic F-127 (Invitrogen) for 30 min at 37°C. After incubation, cells were blocked using FcR-blocking reagent (TONBO) and stained with antibodies (Supplemental table2). Resuspend cells with Ringer solution (155 mM NaCl, 4.5 mM KCl, 1 mM $MgCl_2$, 5 mM HEPES, 5.6 mM D-glucose, 0.025% BSA, 2 mM $CaCl_2$) were stimulated with 10 μg/ml anti-mouse IgM $F(ab)'_2$ (Jackson). Changes in fluorescence were monitored on the LSRFortessa X-20 flow cytometer (BD Biosciences).

Sample preparation

Pre-warmed $CD43^-$ B cells (1×10^6 cells) were stimulated with 0.1 mM Pervanadate for 5 min and then lysed in lysis buffer (10 mM Tris HCl [pH7.4], 150 mM NaCl, 1% [vol/vol] Triton X-100, 0.5 mM EDTA) supplemented with protease inhibitor (Nacalai Tesque) and phosphatase inhibitor (Nacalai Tesque) cocktail. For sorted samples, 2.5×10^5 FO B cells and MZ B cells were stimulated with 10 μg/ml LPS (Sigma-Aldrich), and nuclear fraction was extracted by NE-PER Nuclear and Cytoplasmic Extraction Reagent (Thermo Fisher). For the preparation of pervanadate, H_2O_2 was added to vanadate 15 min before experiments.

	% in B cells			Cell numbers (×10^6)		
	WT	Btk KO	*P value*	WT	Btk KO	*P value*
**Spleen**						
B1a	1.58±0.16	0.07±0.01	**	0.392±0.041	0.005±0.001	**
B2	97.80±0.30	99.77±0.06	***	24.504±3.643	7.279±1.923	**
Immature B	30.74±1.55	35.49±4.28		7.674±0.816	2.640±0.923	**
Transitional 1	8.06±0.64	20.51±2.80	**	2.010±0.218	1.531±0.556	
Transitional 2	10.69±0.29	11.09±1.19		2.682±0.439	0.822±0.278	**
Transitional 3	9.40±0.78	1.70±0.10	**	2.339±0.168	0.123±0.028	****
Mature B	66.02±1.99	63.48±4.20		16.581±2.866	4.583±1.035	**
Follicular B	54.64±1.40	52.51±2.77		13.713±2.320	3.815±0.988	**
Marginal Zone B	5.36±1.01	6.73±2.22		1.327±0.405	0.458±0.020	
**Bone Marrow**						
Pre-Pro B	6.38±0.81	7.33±0.54		0.383±0.01	0.39±0.063	
ProB	16.36±1.34	38.79±2.26	***	1.046±0.13	2.21±0.455	*
PreB	31.67±1.69	16.39±0.70	***	2.019±0.13	0.93±0.140	***
Immature B	14.04±0.13	20.58±0.44	****	0.895±0.09	1.16±0.151	
Recirculated B	27.29±3.19	13.25±1.63	**	1.763±0.37	0.74±0.046	*
**PEC**						
B1a	31.70±0.62	0.63±0.25	****	0.203±0.006	0.001±0.000	***
B1b	16.73±1.68	9.57±2.76	*	0.107±0.010	0.009±0.003	****
B2	49.13±1.99	89.53±3.01	****	0.314±0.014	0.083±0.029	***

Supplemental Table 1. B cell population in Btk KO mice

The number of cells and percentage in B cells were calculated on the basis of total cell count and flow cytometric analysis. Each B cell subsets were distinguished as in Supplemental Figure 3. Data are presented as mean ± SD for three mice. Significant differences are shown (*$p < 0.05$, **$p < 0.01$, ***$p < 0.001$, and ****$p < 0.0001$; using unpaired two-tailed Student's *t*-test or two-tailed Welch's *t*-test).

For flow cytometry analysis			
**Specificity**	**Clone**	**Conjugation**	**Source**
IgG1	RMG1-1	FITC	BioLegend
CD19	6D5	FITC	BioLegend
CD23	B3B4	FITC	BioLegend
CD5	53-7.3	APC	BioLegend
CD19	6D5	APC	BioLegend
CD25	PC61	APC	BioLegend
CD86	GL-1	APC	BioLegend
B220	RA3-6B2	APC-Cy7	BioLegend
CD138	281-2	PE	BD Biosciences
CD80	16-10A1	PE	BioLegend
IgM	RMM-1	PE	BioLegend
CD21/CD35	7E9	PE-Cy7	BioLegend
IgD	11-26c.2a	PerCP-Cy5.5	BioLegend
CD93	AA4.1	BV421	BD Biosciences
CD43	eBioR2/60	Biotin	eBiosciense
CD69	H1.2F3	Biotin	BioLegend
**For cell sorting**			
**Specificity**	**Clone**	**Conjugation**	**Source**
CD3e	145-2C11	FITC	BioLegend
CD11b	M1/70	FITC	BioLegend
CD19	6D5	FITC	BioLegend
NK1.1	OK136	FITC	BioLegend
CD23	B3B4	FITC	BioLegend
CD19	6D5	APC	BioLegend
Siglec-F	S17007L	APC	BioLegend
CD11b	M1/70	APC-Cy7	BioLegend
B220	RA3-6B2	APC-Cy7	BioLegend
CD23	B3B4	PE	BioLegend
F4/80	BM8	PE	BioLegend
CD21/CD35	7E9	PE-Cy7	BioLegend
CD45.2	104	PE-Cy7	BD Biosciences
CD93	AA4.1	BV421	BD Biosciences
Ly6G	1A8	BV421	BioLegend
Siglec-F	S17007L	Biotin	BioLegend
**For Ca^{2+} measurement**			
**Specificity**	**Clone**	**Conjugation**	**Source**
CD23	B3B4	APC	BioLegend
B220	RA3-6B2	APC-Cy7	BioLegend
CD21/CD35	7E9	PE-Cy7	BioLegend

Supplemental Table 2. Antibodies used for flow cytometry.

Abbreviations: FITC, fluorescein isothiocyanate; APC, allophycocyanin; BV, Brilliant Violet; PE, Phycoerythrin; Cy, cyanine; PerCP, Peridinin-Chlorophyll-Protein Complex

gene	Sense primer	Antisense primer
il6	5′- GAGGATACCACTCCCAACAGACC -3′	5′- AAGTGCATCATCGTTGTTCATACA -3′
cxcl10	5′- CTTCTGAAAGGTGACCAGCC -3′	5′- GTCGCACCTCCACATAGCTT -3′
tnf	5′- GAACTGGCAGAAGAGGCACT -3′	5′- AGGGTCTGGGCCATAGAACT -3′
nos2	5′- ACATCGACCCGTCCACAGTAT -3′	5′- CAGAGGGGTAGGCTTGTCTC -3′
β–actin	5′- GCTCTTTTCCAGCCTTC -3′	5′- CGGATGTCAACGTCACA -3′

Supplemental Table 3. Primer for quantitative RT-PCR.

Specificity	Clone	Conjugation	Source
**1st antibody**			
p-Btk (Y223)	D9T6H	-	Cell Signaling Technology
p-PLCγ2	Tyr1217	-	Cell Signaling Technology
p-Erk1/2 (Thr202/Tyr204)	D13.14.E	-	Cell Signaling Technology
Erk1/2	281-2	-	Cell Signaling Technology
p65	D14E12	-	Cell Signaling Technology
Btk	E-9	-	Santa Cruz Biotechnology
Btk	polyclonal	-	Novus Biologicals
PLCγ2	B-10	-	Santa Cruz Biotechnology
Lamin B	C-20	-	Santa Cruz Biotechnology
**2nd antibody**			
Mouse IgG (H+L)	polyclonal	HRP	Invitrogen
Rabbit IgG (H+L)	polyclonal	HRP	Invitrogen

Supplemental Table 4. Antibodies used for western blotting.
Abbreviations: HRP, Horseradish peroxidase

Supplemental figure 1

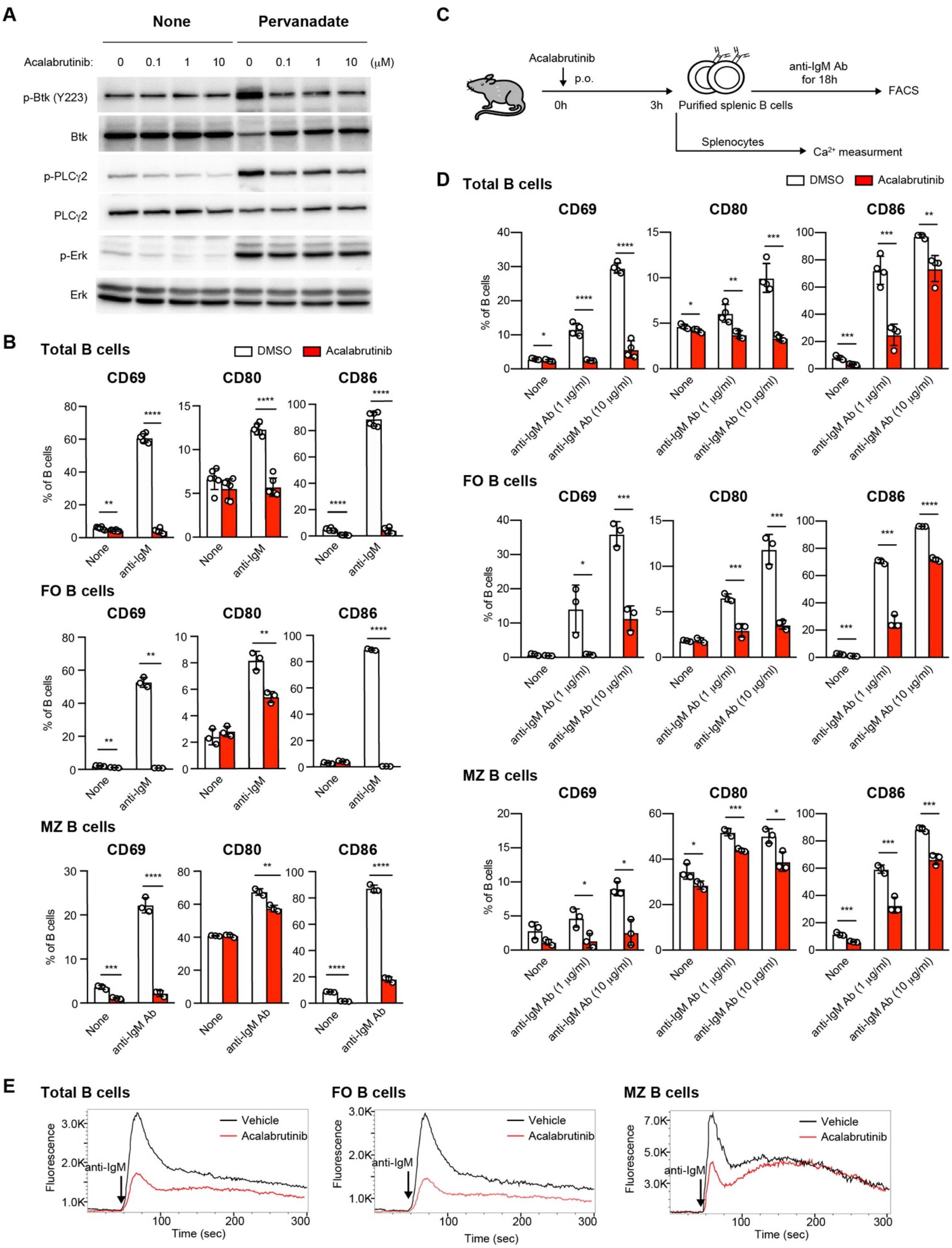

Supplementary Figure 1

Acalabrutinib inhibits Btk Y223 phosphorylation and BCR downstream signaling.

(A) Immunoblot analysis of whole-cell lysates of acalabrutinib pre-treated splenic B cells stimulated with pervanadate for 5 min. p-, phosphorylated (B) Flow cytometric analysis of cell surface expression of CD69, CD80, and CD86 in splenic total B cells, FO B cells, and MZ B cells stimulated with 10 μg/ml anti-IgM Ab for 18 h with vehicle or 1 μM acalabrutinib. (C) Schematic of screen workflow. Mice were given vehicle or 10 mg/kg acalabrutinib by p.o., and then the splenic B cells were analyzed 3 h after administration. (D) Flow cytometric analysis of cell surface expression of CD69, CD80, and CD86 in total B cells, FO B cells, and MZ B cells stimulated with 1 or 10 μg/ml anti-IgM Ab for 18 h after 3 h administration. (E) Ca^{2+}-mobilization profiles in the presence of 2 mM extracellular Ca^{2+} in splenocytes were stimulated with 10 μg/ml anti-IgM Ab 3h after administration, monitored by Cal-520 AM imaging. Total B cell ($B220^{+}$), FO B cell (FO; $B220^{+}$ $CD23^{+}$ $CD21^{int}$) and MZ B cell (MZ; $B220^{+}$ $CD23^{lo}$ $CD21^{hi}$) were gated and analyzed. Data from the bar graph are presented as mean ± SD for six (Total B cells), three (FO B cells and MZ B cells) (B) or four (Total B cells), three (FO B cells and MZ B cells) (D) mice. Data shown are pooled from two independent experiments (Total B cells) (B, D). Representative data are shown of three mice (E). Significant differences are shown ($^{*}p < 0.05$, $^{**}p < 0.01$, $^{***}p < 0.001$, and $^{****}p < 0.0001$; using unpaired two-tailed Student's *t*-test or two-tailed Welch's *t*-test).

Supplemental Figure 2

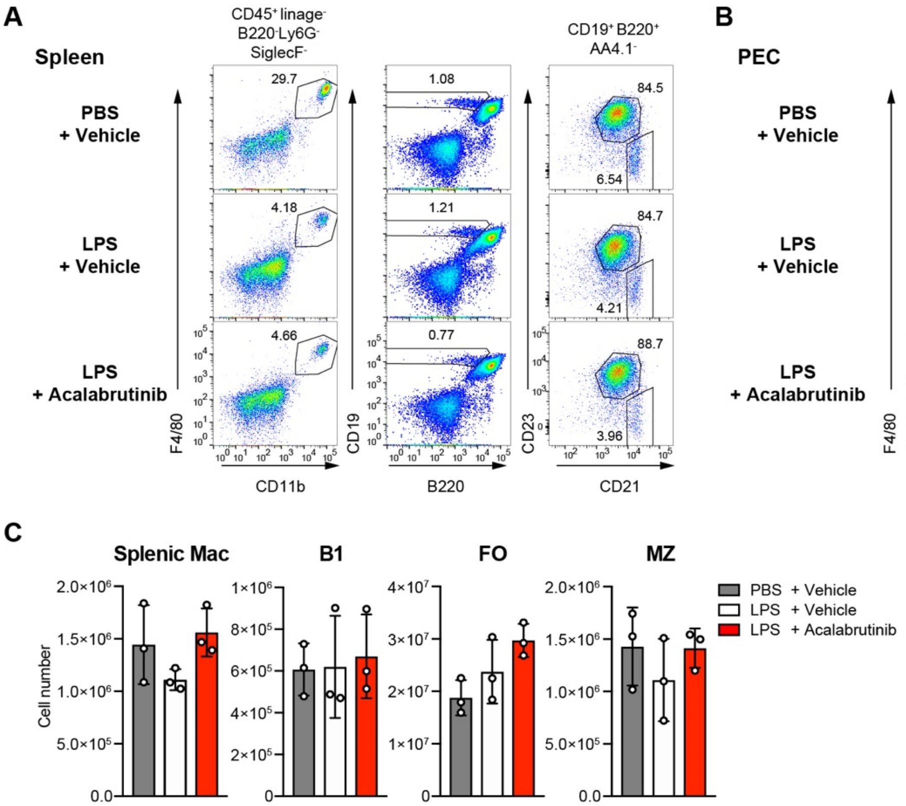

Supplementary Figure 2. Sorting strategy and effect of acalabrutinib on B cells and macrophages.

The sorting strategy is shown for the same experimental condition as in Figure 1. (A) Dot plots are shown a gating strategy to identify as follow: splenic Mac; CD45^{+} lineage^{-} (CD3ε^{-} NK1.1^{-} CD19^{-}) B220^{-} Ly6G^{-} siglecF^{-} F4/80^{+} CD11b^{+}), B1 B cell (B1; CD19^{+}B220$^{lo/-}$), FO B cell (FO; CD19^{+} AA4.1^{-} B220^{+} CD23^{+} CD21^{int}), MZ B cell (MZ; CD19^{+} AA4.1^{-} B220^{+} CD23^{lo} CD21^{hi}). (B) Dot plots are shown a gating strategy to identify as follow: PEC, macrophage (PEC Mac; CD45^{+} Ly6G^{-} siglecF^{-} F4/80^{+} CD11b^{+}) in PEC. (C) Bar graphs show the means ± SD for three mice of cell numbers in splenic Mac, B1, FO, and MZ. Data are representative of three independent experiments.

Supplemental Figure 3

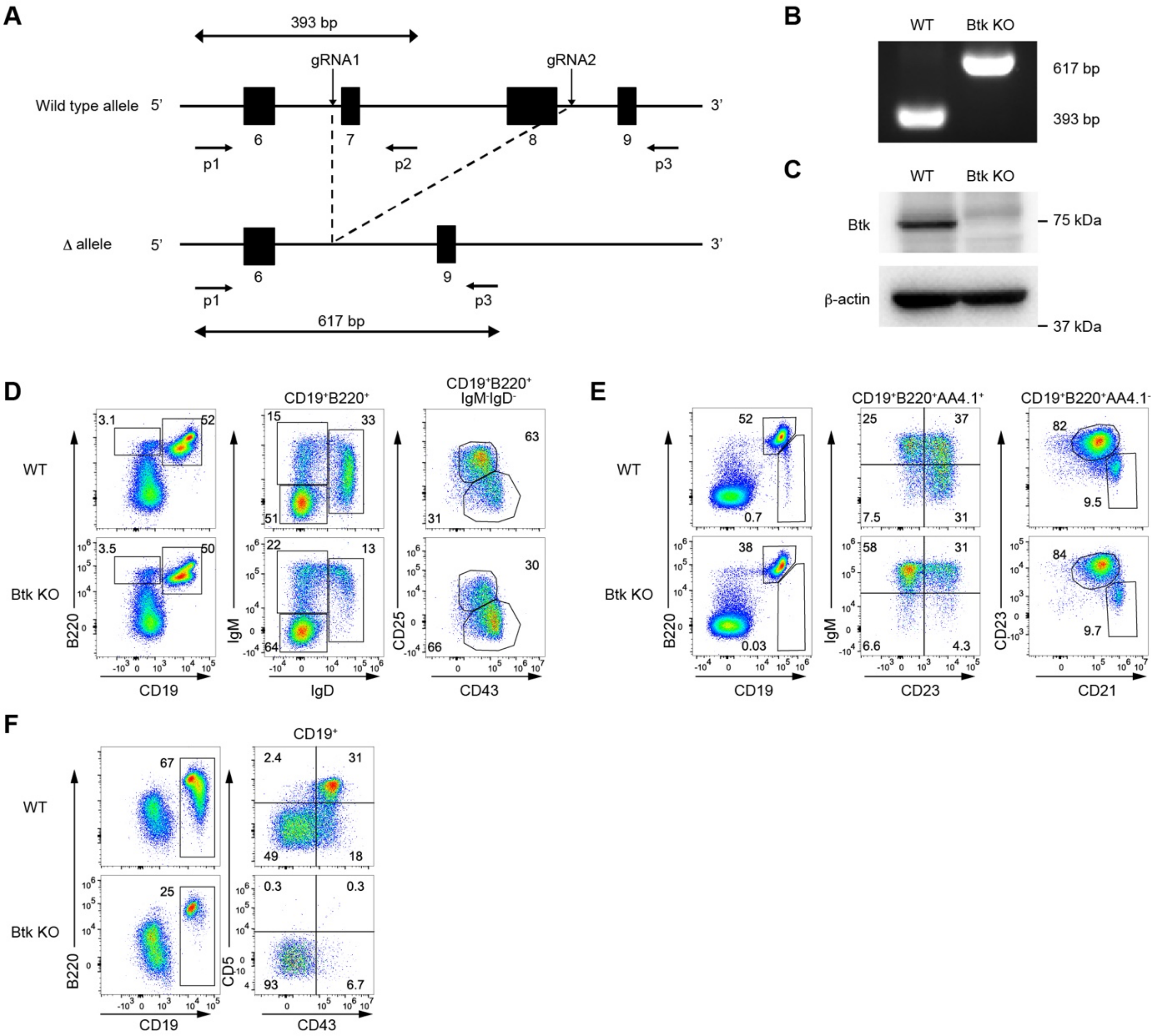

Supplementary Figure 3. B cell development in Btk KO mice.

(A) Schematic representation of the targeting strategy and genotyping strategy. Targeting gRNAs was designed between exon6 and exon7; gRNA1, and between exon8 and exon9; gRNA2. (B) PCR for detection of wild-type (WT) and deletion (Δ) allele with genomic DNA from WT and Btk KO mice. Amplicons of 393 and 617 bp are identified by primer pairs (p1-p2 and p1-p3) specific for the WT and Δ alleles, respectively. (C) Immunoblot analysis of whole-cell lysates of bone marrow cells from WT and Btk KO mice. (D-F) Flow cytometry of each B cell subset from BM (D), spleen (E), and PEC (F) from WT and Btk KO mice. Representative data are shown of three mice per group (B-F).

Supplemental Figure 4

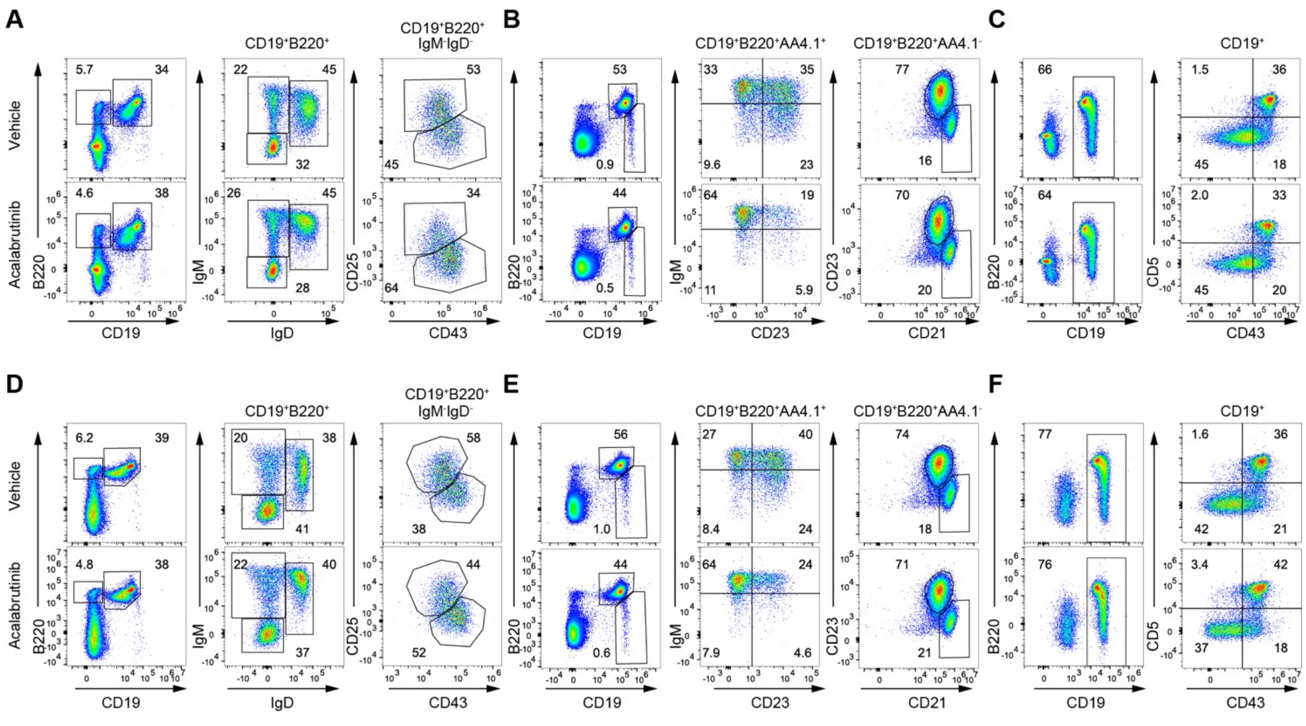

Supplementary Figure 4. The effect of acalabrutinib on B cell development.

Flow cytometry of each B cell subset from BM (A, D), spleen (B, E), and PEC (C, F) for the same experimental condition as in Figure 4. Vehicle (2% HPBCD) or 0.15 mg/ml acalabrutinib were administrated by drinking water for 1 week (A-C) or 3 weeks (D-F). Representative data are shown of two independent experiments (A-F).
